# Influence of Fluorine Substituents on the Electronic Properties of Selenium-*N*-Heterocyclic Carbene Compounds

**DOI:** 10.3390/molecules25215161

**Published:** 2020-11-06

**Authors:** Mohamad Shazwan Shah Jamil, Nor Azam Endot

**Affiliations:** 1 Department of Chemistry, Faculty of Science, Universiti Teknologi Malaysia, Johor Bahru 81310 UTM, Johor, Malaysia; 2 Department of Chemistry, Faculty of Science, Universiti Putra Malaysia, Serdang 43400 UPM, Selangor, Malaysia

**Keywords:** *N*-heterocyclic carbene, selenium, fluorine, NMR spectroscopy

## Abstract

*N*-heterocyclic carbenes (NHCs) are common ancillary ligands in organometallic compounds that are used to alter the electronic and steric properties of a metal centre. To date, various NHCs have been synthesised with different electronic properties, which can be done by modifying the backbone or changing the nitrogen substituents group. This study describes a systematic modification of NHCs by the inclusion of fluorine substituents and examines the use of selenium-NHC compounds to measure the π-accepting ability of these fluorinated NHC ligands. Evaluation of the ^77^Se NMR chemical shifts of the selenium adducts reveals that fluorinated NHCs have higher chemical shifts than the non-fluorinated counterparts, IMes and IPh. Higher ^77^Se NMR chemical shifts values indicate a stronger π-accepting ability of the NHC ligands. The findings of this study suggest that the presence of fluorine atoms has increased the π-accepting ability of the corresponding NHC ligands. This work supports the advantage of the ^77^Se NMR chemical shifts of selenium-NHC compounds for assessing the influence of fluorine substituents on NHC ligands.

## 1. Introduction

*N*-Heterocyclic Carbenes (NHCs) are excellent spectator ligands which contribute to many applications in homogenous transition metal catalysis, such as olefin metathesis [1,2], cross-coupling [3,4,5,6] and asymmetric catalysis [7]. Preliminary work on NHCs began as early as 1835 but attracted more attention in 1968 when Wanzlick and Ofele independently synthesised the first NHC-metal complexes, 1,3-bis(adamantyl)imidazol-2-ylidene bearing mercury(II) and chromium(0), respectively [8,9,10]. Subsequently, the progress of NHCs was dormant because carbenes were very unstable and could not be isolated. In 1991, Arduengo et al., made a breakthrough when they reported the successful isolation and characterisation of an NHC, which led to the rapid development in this field with a range of NHCs being synthesised and analysed [11]. 

**Figure 1:**
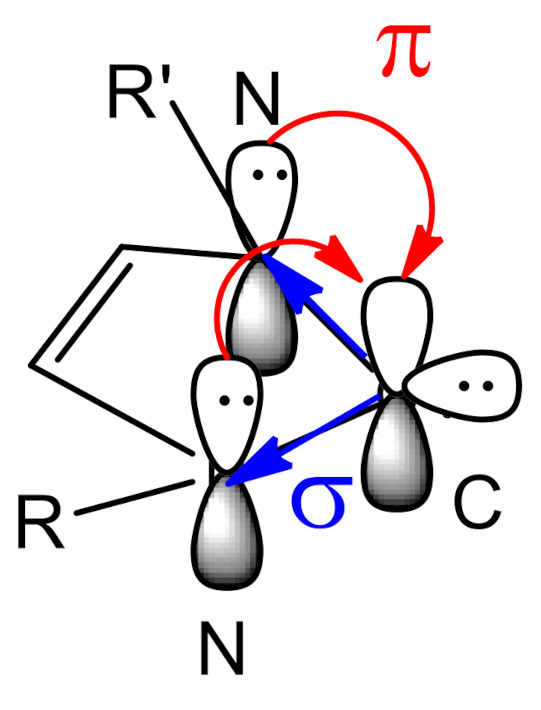
σ and π interactions between carbene and adjacent nitrogen atoms.

NHCs are good electron donors due to the presence of nitrogen atoms in the ring, which can provide electronic stabilisation to the metal compound. Non-bonding electrons on the carbene can exist in paired (singlet) or unpaired (triplet) states. Most NHCs exhibit a singlet ground-state electronic configuration with the highest occupied molecular orbital (HOMO) being a *sp*^2^-hybridised lone pair of the carbene whilst the lowest unoccupied molecular orbital (LUMO) is described as an unoccupied *p*-orbital of the carbene. Nitrogen atoms provide both inductive and mesomeric stabilisation to the carbene through σ-electron-withdrawing and π-electron-donating by lowering the energy of the occupied σ-orbital and donating electron density into the empty *p*-orbital respectively. The presence of nitrogen atoms, which are π-donors, increases the nucleophilicity of the carbene, which favours the singlet state by forcing the carbene carbon into a bent, more *sp*^2^-like arrangement as shown in Figure 1. Changing the substituent groups (R, R′) on the nitrogen atoms will affect the electronic properties of NHCs.

**Figure 2:**
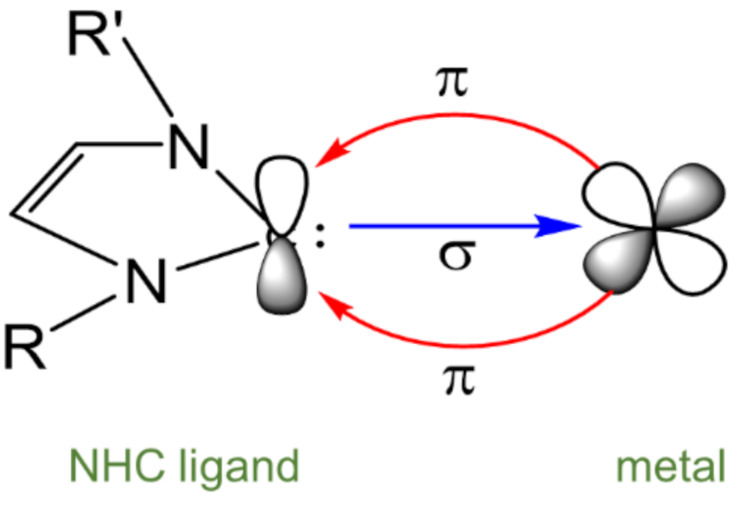
σ and π interactions of an NHC and a transition metal.

The bonding between NHCs and transition metals consist of σ and π interactions. NHCs contain a formal *sp*^2^-hybridised lone pair in the carbene *p*-orbital which is available for σ-donation to an empty *d*-orbital of a transition metal. In addition, π back donation can occur from a filled metal orbital to the empty orbital of π symmetry on the carbene. These two interactions are portrayed in Figure 2 below.

**Figure 3:**
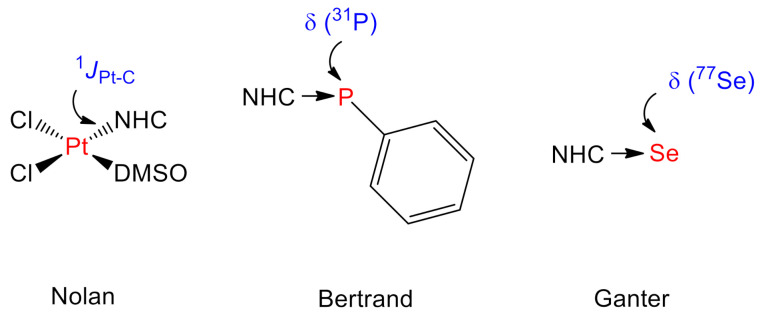
Techniques developed to quantify the π-acceptor properties of NHCs as proposed by Nolan, Bertrand and Ganter [14,15,16].

Comparing these two interactions, σ-donation is the key and predominant component of the bonding between an NHC and a metal. Several reports have discussed in detailed the measurement and evaluation of σ-interaction, for example by investigating the carbonyl stretching frequencies in [Ni(CO)_3_(NHC)] and [IrCl(CO)_2_(NHC)] [12,13]. However, these values from Infra-Red (IR) spectroscopy does not provide an insight into the relative contribution of π-acceptor parameters of a particular NHC. To overcome this lack of information, a number of alternative methods using NMR spectroscopies have been proposed to determine the π-accepting ability of NHCs from the metal centre (Figure 3)Nolan utilised the platinum active nuclei to analyse the ^1^*J*_Pt-C_ coupling constants in [PtCl_2_(DMSO)(NHC)] complexes [14], while Bertrand studied the ^31^P NMR chemical shifts of phosphinidene adducts [15]. Subsequently, Ganter proposed the use of ^77^Se NMR chemical shift of selenium adducts and suggested that increasing π-acidity of NHCs would lead to a downfield shift of the selenium signal [16]. Amongst these methods, the selenium system has more advantages than the phosphinidene and platinum systems. Selenium adducts can be prepared in a one-step reaction from the air-stable NHC precursors and selenium whilst phosphinidene adducts require two-step procedures from the air-sensitive free NHCs. In addition, selenium precursors are cheap and metal-free compared to platinum complexes which are more expensive and involved multi-step preparation. Furthermore, in term of practicality and feasibility in acquiring and analysing the NMR spectra, the selenium system is more convenient than carbon and proton systems. The receptivity of ^77^Se nuclei is relatively much higher than ^13^C nuclei (7.5% and 1.1% natural abundances respectively). This means that for ^77^Se system, spectra with a reasonable signal-to-noise ratio can be obtained within a reasonable time (30−60 min) [16]. However, in the case of ^13^C system, a much longer time is required to run the samples in order to acquire good spectra [17]. Besides, selenium lacks additional substituents apart from the NHC, hence only one single resonance will appear on their spectra. On the other hand, ^1^H and ^13^C NMR spectra will show multiple signals due to various substituents and chemical environments which made the signal assignment more complicated [18].

NHCs coordinated to transition metals are excellent for catalysis due to their electronic properties. The presence of fluorine in an NHC is likely to impact to some extent the electron donor and acceptor abilities. Theoretical and computational studies have shown that π interaction contributes a significant impact on the bonding between a carbene and transition metal [19,20]. In these reports, they stated that the presence of a fluorinated group on the *N*-substituents can reduce the energy of the LUMO hence increasing their π-accepting ability from π-back bonding of metals. This results in a stronger NHC-metal bond and reducing the electron density on the metal of the NHC-metal complexes [21]. The π-back bonding interaction in the metal-NHC complexes involves π-donation from the metal centre into the π-accepting LUMO of NHC. Generally, the LUMOs of free NHCs are quite high in energy, relative to other kinds of carbenes. This is why NHCs are strong σ-donors but relatively weak π-acceptors. However, the substituents on nitrogen do influence the electronic properties of NHCs, particularly in the π-accepting LUMO of NHCs. By introducing a fluorinated group on the *N*-substituents, it could lower the energy of the LUMO of NHCs, resulting in better π-accepting ability from the metal π-back bonding orbital.

Inspired by the excellent use of ^77^Se NMRspectroscopy, we aim to investigate the influence of fluorine substituents on the electronic properties of selenium-NHC adducts by evaluating their π-accepting ability from the ^77^Se NMR chemical shifts. This will provide some information on whether the presence of fluorine will affect the electronic properties of NHCs. The data can be compared with the non-fluorinated analogues that have been reported before.

## 2. Results and Discussion

**Scheme 1:**
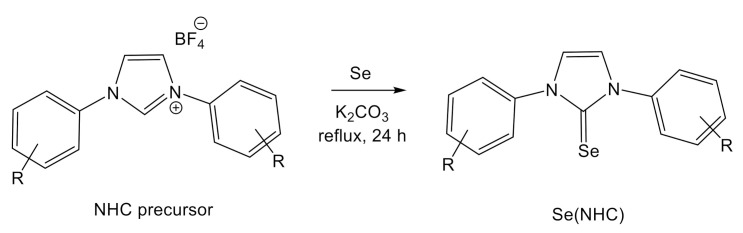
Schematic diagram for the preparation of selenium-NHC adducts.

In order to complement the theoretical studies, experimental works must be carried out to evaluate the π-accepting ability of the NHCs containing fluorinated substituent groups. In this study, a series of selenium adducts featuring fluorinated NHCs and non-fluorinated analogues were prepared according to the literature procedure with a slight modification. A previous report described the synthesis of selenium-NHC adducts under argon atmosphere in a glove box [22]. However, in this study, the selenium-NHC adducts were prepared in air. This is because the NHC precursors (in the form of imidazolium salts) were found stable to air and moisture. The appropriate fluorinated **NHC-1** to **NHC-5** precursors and the non-fluorinated analogues, IMes and IPh precursors were reacted with excess selenium, in the presence of potassium carbonate as shown in Scheme 1. All compounds were air and moisture-stable and fully characterised by multinuclear NMR spectroscopies and elemental analysis. 

**Figure 4:**
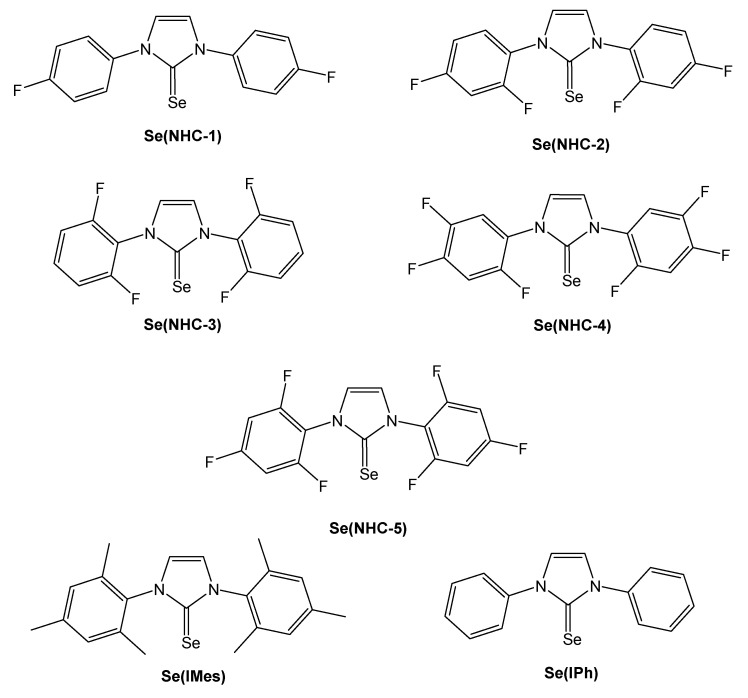
Selenium-NHC adducts prepared in this study; those containing fluorinated NHCs {**Se(NHC-1)**–**Se(NHC-5)** and the non-fluorinated analogues, **Se(IMes)** and **Se(IPh).**

Figure 4 shows the selenium adducts of fluorinated NHCs {**Se(NHC-1)**–**Se(NHC-5)**} and the non-fluorinated NHC precursors, IMes and IPh. The compounds **Se(NHC-1)** to **Se(NHC-5)** feature fluorine substituents with different number and position of fluorine atoms whilst **Se(IMes)** possesses methyl substituents in the *ortho* and *para* positions of the phenyl rings. On the other hand, **Se(IPh)** does not have any substituents in the aromatic ring of the nitrogen substituents.

Having successfully synthesised and characterised the selenium-NHC adducts, their ^77^Se NMR chemical shifts were recorded in deuterated chloroform(chloroform-*d*) and spanned in a range from 24 to 73 ppm. ^77^Se NMR spectra were referenced to external (CH_3_)_2_Se in chloroform-*d* with a chemical shift of −4.86 ppm. In addition to the chemical shifts, Ganter et al., extended the use of ^77^Se NMR spectroscopy to assess the σ-donor strength of various NHC ligands, by measuring the coupling constant of the carbene and selenium, ^1^*J*_CSe_ [23]. In NMR spectroscopy, it is understood that the coupling constants between directly bonded atoms are due to the interaction of the electron density in *s* orbitals between the neighbouring atoms. Previous studies have shown that the coupling constant between phosphorus and selenium of the selenium-phosphine adducts arise from the basicity of the corresponding phosphine [16,17,18]. Strong electron-withdrawing substituents of a phosphine ligand would increase the *s* character of the phosphorus orbital involved in bonding to the selenium atom, leading to a higher coupling constant between the phosphorus and selenium nuclei. Hence, decreasing basicity of NHC ligand would result in an increase in the coupling constant. Taking advantage of this technique, we have attempted to obtain the coupling constants, ^1^*J*_CSe_, from the selenium satellite signals in the ^13^C{^1^H} NMR spectra. However, all the attempts were unsuccessful due to the low natural abundances of ^13^C and ^77^Se, which are only 1.1 and 7.5% respectively. The chance for both of these nuclei being spin-active in a single molecule is very slim, with just 0.08% probability of this occurring. This problem has also been faced previously by Ganter and co-workers, as described in their work [23].

Table 1 tabulates the percentage yield and ^77^Se NMR chemical shift data of the selenium adducts prepared in this work. Higher ^77^Se NMR chemical shifts values correlate to lower field resonances due to a less shielded selenium nucleus, which indicate a stronger π-accepting ability of the NHC ligands. According to Table 1, selenium adducts containing fluorinated NHC ligands, **Se(NHC-1)** to **Se(NHC-5)** have higher ^77^Se NMR chemical shifts values than the non-fluorinated analogues, **Se(IMes)** and **Se(IPh)**. Based on this finding, the presence of fluorine atoms in the NHC ligands has increased the π-accepting ability of the NHC ligands. A possible explanation for this finding may be due to the strong electron-withdrawing effect of the fluorine substituents in the NHC ligands. This, in turn, generate more electron-deficient carbenes which increase the π-acceptor strength from the neighbouring selenium. The findings from this work are consistent with a previous theoretical study which demonstrated that the presence of fluorine in the nitrogen substituent groups could lower the energy of the LUMO, hence improving the accepting ability of an NHC from π-back bonding of metal [21].

Amongst the fluorinated NHC selenium adducts **Se(NHC-1)** to **Se(NHC-5)**, those with the higher number of fluorine substituents, such as **Se(NHC-4**) and **Se(NHC-5**) possess the strongest π-acceptor strength compared with the lower fluorine-content analogues **Se(NHC-1)** and **Se(NHC-2)**. Surprisingly, **Se(NHC-3)** that contains only two fluorine substituents has a higher π-accepting ability than its greater fluorine content counterpart, **Se(NHC-4)**. Consideration of the structure of these three compounds reveals that the position of fluorine substituents in the aromatic ring of the nitrogen substituents also has an impact to the electronic properties of a particular NHC, apart from the number of fluorine substituents. **Se(NHC-3)** possesses four fluorine substituents in the ortho positions of the aromatic rings of the nitrogen substituents. This position is the closest to the nitrogen of the heterocycle ring and thus having fluorine substituents in this position appears to increase the π-accepting ability of the NHC ligands, as indicated by the ^77^Se NMR chemical shift values.

**Figure 5:**
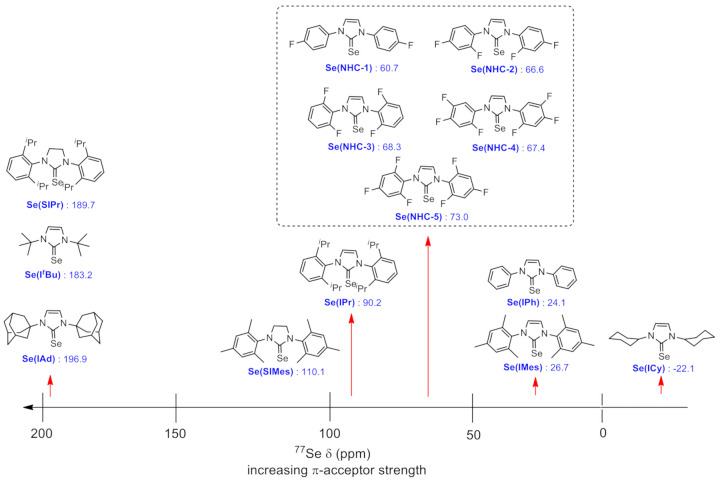
The ^77^Se NMR chemical shifts for various selenium-NHC compounds.

Figure 5 displays the ^77^Se NMR chemical shift range for selenium adducts containing various imidazole and imidazolidene NHC type ligands. Generally, saturated NHC ligands (imidazolidene) such as SIPr and SIMes are more π-accepting than the unsaturated (imidazole) NHCs ligands, IPr and IMes. This is reflected by the higher values of the ^77^Se NMR chemical shift, 189.7 ppm for SIPr and 110.1 ppm for SIMes than their counterparts, 90.2 ppm and 26.7 ppm for IPr and IMes respectively. Surprisingly, there are no studies involving selenium adducts of the fluorinated NHC ligands reported so far. Therefore, the results obtained from this study offer some important insights into the electronic parameters of the fluorinated NHC ligands and provide more information on how the presence of fluorines may influence the electronic properties of NHC ligands.

**Figure 6:**
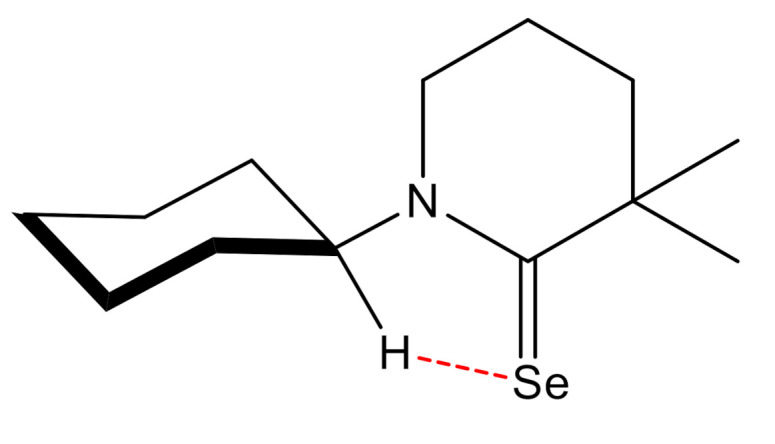
The intramolecular Se-H interaction observed in the CAAC-selenium adduct.

Although this concept provides a good measure of π-acceptor strength of the NHC ligands, however, it must be taken into accounts that there is a limitation when it comes to very bulky ligands. The steric bulk of a ligand can always outweigh the electronic characters. For example, binding of a bulky ligand could be difficult due to steric hindrance despite favourable electronic interactions [17]. This phenomenon can be exemplified by comparing the ^77^Se NMR chemical shifts of the Se(IAd) and **Se(ICy)** presented in Figure 5. Despite having similar structures, the ^77^Se NMR chemical shifts of **Se(IAd)** and **Se(ICy)** appear in the opposite extremes with the value of 196.9 and −22.1 ppm respectively. Initially, one might predict that their electronic properties should be similar, however the steric differences between the IAd and ICy ligands may cause variation in their electronic properties. This disparity observation has been further supported by Bertrand et al. in their recent work involving a family of cyclic alkly(amino) carbenes (CAAC)-selenium adducts [24]. The findings from experimental and computational works demonstrated the existence of non-classical hydrogen bonding (NCHB), arising from a negative hyperconjugation interaction between the lone pair of selenium (H-bond acceptor) and the σ*_C-H_ orbital of C(*sp*^3^)-H (H-bond donor), as shown in Figure 6. This additional interaction causes deshielding of selenium nucleus and results in a larger chemical shift. Although their work did not involve the NHC family of imidazolium system, Bertrand and co-workers proposed that the same reasoning can also be applied to imidazolium systems, especially when large substituents are in close distance to the carbene centre. Ultimately, both the electronic and steric properties are two important characteristics (hence the term stereoelectronic) in the interactions between the NHC ligands and the metal/selenium and these properties cannot be treated separately [18]. Therefore, it is necessary to use this technique carefully and understand the scope and limitations of this technique for measuring the π-acceptor strength of the NHC ligands.

## 3. Materials and Methods

### 3.1. General Considerations

All reactions were carried out in air. NHC precursors, **NHC-1**, **NHC-2**, **NHC-3**, **NHC-4**, **NHC-5**, IMes and IPh precursors were prepared according to the published procedure [18,25]. Fluoroanilines, selenium and potassium carbonate were purchased from Fluorochem. All other chemicals were obtained commercially from Sigma Aldrich or Alfa Aesar and were of analytical grade or higher and were used without further purification. ^1^H, ^13^C, ^19^F and ^77^Se NMR spectra were recorded using a Bruker Avance 400 MHz spectrometer, and were referenced to external TMS (^1^H, ^13^C) and CFCl_3_ (^19^F) and (CH_3_)_2_Se (^77^Se). Chemical shifts (δ) are reported in parts per million (ppm) and coupling constants (J) are reported in Hertz (Hz). Elemental analyses for carbon, hydrogen and nitrogen were performed on Thermo Scientific Flash 2000 Organic Elemental Analyser.

#### 3.1.1. 1,3-Bis(4-fluorophenyl)imidazol-2-selenone, **Se(NHC-1)**

In a 100 mL round bottom flask, **NHC-1** precursor (0.580 g, 1.70 mmol), selenium powder (0.135 g, 1.70 mmol) and potassium carbonate (0.290 g, 2.10 mmol) were mixed in methanol (50 mL) and heated at reflux for 24 h. After this time, the mixture was concentrated on a rotary evaporator to remove the solvent. The crude product was dissolved in dichloromethane (30 mL), then the solution was filtered through celite for purification. The filtrate was concentrated on a rotary evaporator to remove the solvent and the resulting solid was recrystallized from dichloromethane (5 mL) and pentane (20 mL) mixture and filtered, to afford an off-white solid (0.50 g, 81%). ^1^H NMR (CDCl_3_) δ (ppm): 7.61 (s, 2H, NCHCHN), 7.20 (m, 4H, ^3^*J*_HH_ = 4.6 Hz, H_Ar_), 7.12 (m, 4H, ^3^*J*_HH_ = 8.9 Hz, H_Ar_). ^13^C{^1^H} NMR (CDCl_3_) δ (ppm): 163.39, 134.87, 128.70, 123.90, 120.99, 116.30. ^19^F{^1^H} NMR (CDCl_3_) δ (ppm): −111.60 (s, 2F, F). ^77^Se{^1^H}NMR (CDCl_3_) δ (ppm): 60.7. Anal. Calcd for C_15_H_10_F_2_N_2_Se (%): C, 53.72, H, 3.01, N, 8.36. Found: C, 53.48, H, 3.43, N, 8.69. 

#### 3.1.2. 1,3-Bis(2,4-difluorophenyl)imidazol-2-selenone, **Se(NHC-2)**

A synthetic method similar to that used for **Se(NHC-1)** afforded an off-white solid (0.520 g, 82%). ^1^H NMR (CDCl_3_) δ (ppm): 8.20 (s, 2H, NCHCHN), 7.72 (m, 2H, H_Ar_), 7.31 (s, 2H, H_Ar_), 7.19 (m, 2H, H_Ar_). ^13^C{^1^H} NMR (CDCl_3_) δ (ppm): 163.40, 155.82, 149.91, 131.13, 129.85, 121.32, 112.19, 105.16. ^19^F{^1^H} NMR (CDCl_3_) δ (ppm): −106.15 (d, 2F, ^4^*J*_FF_ = 8.5 Hz, 4-F_Ar_), −115.35 (d, 2F, ^4^*J*_FF_ = 8.5 Hz, 2-F H_Ar_). ^77^Se{^1^H} NMR (CDCl_3_) δ (ppm): 66.7. Anal. Calcd for C_15_H_8_F_4_N_2_Se (%): C, 48.50, H, 2.14, N, 7.60. Found: C, 48.50, H, 2.41, N, 7.68.

#### 3.1.3. 1,3-Bis(2,6-difluorophenyl)imidazol-2-selenone, **Se**(**NHC-3)**

A synthetic method similar to that used for **Se(NHC-1)** produced an off-white solid (0.535 g, 84%). ^1^H NMR (CDCl_3_) δ (ppm): 8.29 (s, 2H, NCHCHN), 7.54 (s, 2H, H_Ar_), 7.21 (t, 4H, ^3^JHH = 8.8 Hz, H_Ar_). ^13^C{^1^H} NMR (CDCl_3_) δ (ppm): 164.68, 153.30, 141.19, 131.59, 122.26, 112.30. ^19^F{^1^H} NMR (CDCl_3_) δ (ppm): −118.7 (s, 4F, F_Ar_). ^77^Se{^1^H} NMR (CDCl_3_) δ (ppm): 68.3. Anal. Calcd for C_15_H_8_F_4_N_2_Se (%): C, 48.61, H, 2.18, N, 7.54. Found: C, 48.71, H, 2.13, N, 7.49.

#### 3.1.4. 1,3-Bis(2,4,5-trifluorophenyl)imidazol-2-selenone, **Se(NHC-4)**

Using a similar synthetic method to that used for **Se(NHC-1)** afforded an off-white solid (0.577 g, 83%). ^1^H NMR (CDCl_3_) δ (ppm): 8.30 (s, 2H, NCHCHN), 7.83 (m, 2H, H_Ar_), 7.70 (s, 2H, H_Ar_). ^13^C{^1^H} NMR (CDCl_3_) δ (ppm): 152.66, 149.36, 145.19, 136.62, 122.36, 117.57, 114.61, 106.21. ^19^F{^1^H} NMR (CDCl_3_) δ (ppm): −125.20 (dd, 2F, ^4^*J*_FF_ = 14.7 Hz, ^5^*J*_FF_ = 5.2 Hz, 2-F_Ar_), −127.20 (dd, 2F, ^3^*J*_FF_ = 23.3 Hz, ^5^*J*_FF_ = 5.2 Hz, 5-F_Ar_), −137.76 (dd, 2F, ^3^*J*_FF_ = 23.2 Hz, ^4^*J*_FF_ = 14.7 Hz, 4-F H_Ar_). ^77^Se{^1^H} NMR (CDCl_3_) δ (ppm): 67.4. Anal. Calcd for C_15_H_6_F_6_N_2_Se (%): C, 44.33, H, 1.50, N, 6.78. Found: C, 44.35, H, 1.60, N, 6.80.

#### 3.1.5. 1,3-Bis(2,4,6-trifluorophenyl)imidazol-2-selenone, **Se(NHC-5)**

A synthetic method similar to that used **Se(NHC-1)** produced an off-white solid (0.584 g, 84%). ^1^H NMR (CDCl_3_) δ (ppm): 8.15 (s, 2H, NCHCHN), 6.90 (m, 4H, H_Ar_). ^13^C{^1^H} NMR (CDCl_3_) δ (ppm): 165.52, 161.80, 157.50, 122.33, 112.50, 101.79. ^19^F{^1^H} NMR (CDCl_3_) δ (ppm): −102.86 (t, 2F, ^4^*J*_FF_ = 7.5 Hz, 4-F_Ar_), −112.65 (d, 4F, ^4^*J*_FF_ = 7.5 Hz, 2,6-F_Ar_). ^77^Se{^1^H} NMR (CDCl_3_) δ (ppm): 73.0. Anal. Calcd for C_15_H_6_F_6_N_2_Se (%): C, 44.33, H, 1.40, N, 6.90. Found: C, 44.41, H, 1.37, N, 6.88.

#### 3.1.6. 1,3-Bis(trimethylphenyl)imidazol-2-selenone, **Se(IMes)**

Using a synthetic method similar to that used for **Se(NHC-1)** produced a white solid (0.535 g, 82%). ^1^H NMR (CDCl_3_) δ (ppm): 7.25 (s, 4H, H_Ar_), 6.98 (s, 2H, NCHCHN), 2.37 (s, 6H, H_Ar_), 2.10 (s, 12H, H_Ar l_). ^13^C{^1^H} NMR (CDCl_3_) δ (ppm): 158.52, 140.90, 135.72, 134.35, 129.11, 120.43, 21.15, 18.60. ^77^Se{^1^H} NMR (CDCl_3_) δ (ppm): 26.7. Anal. Calcd for C_21_H_24_N_2_Se (%): C, 65.75, H, 6.35, N, 7.35. Found: C, 65.81, H, 6.38, N, 7.39.

#### 3.1.7. 1,3-Bis(phenyl)imidazol-2-selenone, **Se(IPh)**

Using a synthetic method similar to that used for **Se(NHC-1)** produced a white solid (0.370 g, 75%). ^1^H NMR (CDCl_3_) δ (ppm): 8.07 (s, 2H, NCHCHN), 7.80 (d, 4H, ^3^*J*_HH_ = 7.6 Hz, H_Ar_), 7.65 (t, 4H, ^3^*J*_HH_ = 7.8 Hz, H_Ar_), 7.52 (t, 2H, ^3^*J*_HH_ = 7.4 Hz, H_Ar_). ^13^C{^1^H} NMR (CDCl_3_) δ (ppm): 155.60, 134.55, 130.22, 130.15, 122.10, 121.82. ^77^Se{^1^H} NMR (CDCl_3_) δ (ppm): 24.1. Anal. Calcd for C_15_H_12_N2Se (%): C, 60.29, H, 4.05, N, 9.30. Found: C, 60.30, H, 4.03, N, 9.28.

## 4. Conclusions

A series of selenium-NHC compounds containing fluorinated NHCs, **Se(NHC-1)** to **Se(NHC-5)** and non-fluorinated analogues **Se(IMes)** and **Se(IPh)** were successfully prepared and characterised. The electronic properties of **NHC-1** to **NHC-5** were assessed by ^77^Se NMR spectroscopy. Evaluation of the ^77^Se NMR chemical shifts of the selenium adducts reveals that these fluorinated NHCs have stronger π-accepting abilities than the non-fluorinated counterparts, IMes and IPh. Generally, the π-accepting ability of NHC ligands increases as the fluorine content in the nitrogen substituent groups increases. The compounds **NHC-3** and **NHC-5** that contain fluorines in the *ortho* position have the greatest influence on the ^77^Se NMR data, relative to the other fluorinated NHC ligands prepared in this work. The number and substitution pattern of fluorine atoms in the substituent groups have significant effects on the electronic properties of NHC ligands. The findings of this study suggest that the presence of fluorine atoms in the NHC ligands has increased the π-accepting ability of NHC ligands. This work contributes to existing knowledge by providing the measurement of the electronic properties of fluorinated NHC ligands and further supports the advantage of the ^77^Se NMR chemical shifts for assessing the electronic parameters of NHC ligands.

## Figures and Tables

**Table 1:** Percentage yields and ^77^Se NMR chemical shift data of selenium-NHC adducts.

Entry	NHC Precursor	Compound	Yield (%)	δ ^77^Se (ppm)
1	**NHC-1**	**Se(NHC-1)**	79	60.7
2	**NHC-2**	**Se(NHC-2)**	81	66.6
3	**NHC-3**	**Se(NHC-3)**	85	68.3
4	**NHC-4**	**Se(NHC-4)**	82	67.4
5	**NHC-5**	**Se(NHC-5)**	83	73.0
6	**IMes**	**Se(IMes)**	81	26.7
7	**IPh**	**Se(IPh)**	68	24.1
